# Supplementary material for: Content-rich biological network constructed by mining PubMed abstracts
Source: BMC Bioinformatics. 2004 Oct 8;5:147. doi: 10.1186/1471-2105-5-147 (PMC528731; doi:10.1186/1471-2105-5-147)
Supplement: Additional File 2 — The original results of the above study (non-essential files are deleted to keep the file size under the limit set by BMC bioinformatics). [file 1471-2105-5-147-S2.bz2 › chilibotAdditionalFile2/dip10/606ID10207005E10988/html/LIFR_CNTF.html]

 


 **LIFR** and **CNTF** 
  
Found 72 abstracts in PubMed, retrieved 10.  
 

 What does Google say? 
 PDF only 
| .edu only 

---

**Interactive relationship** (e.g. stimulation, inhibition, etc)

**Inhibitory relationship**- The CDFs leukemia inhibitory factor and ciliary neurotrophic factor  [ **CNTF** ]  bind to a  **LIFR**  gp130 receptor complex to activate Jak signal transducers and activators of transcription and Ras mitogen activated protein kinases signaling pathways.  Ref: 12609984 J Biol Chem< MedlineTA>J Biol Chem,
- The functional receptor complex of ciliary neurotrophic factor  [ **CNTF** ]   **CNTF** , a member of the gp130 family of cytokines, is composed of  **CNTF** , the  **CNTF**  receptor alpha CNTFR, gp130 and the leukemia inhibitory factor receptor  [ **LIFR** ]   **LIFR** .  Ref: 12707266 J Biol Chem< MedlineTA>J Biol Chem,
**Neutral relationship**- Here, we demonstrate that human  **CNTF**  cannot directly induce a heterodimer of human gp130 and  **LIFR** .  Ref: 12643274 J Biol Chem, 2003
- Here, we demonstrate that human  **CNTF**  cannot directly induce a heterodimer of human gp130 and  **LIFR** .  Ref: 12517918 J Biol Chem< MedlineTA>J Biol Chem, 2003
- Signal transduction by  **CNTF**  requires that it bind first to  **CNTF**  R, permitting the recruitment of gp130 and LIF R  [ **LIFR** ] , forming a tripartite receptor complex.  Ref: 12765290 J Zhejiang Univ Sci< MedlineTA>J Zhejiang Univ Sci, 2003
- These data suggest that both the gp130 CBD and the  **LIFR**  CBD1 interact with CNTFR in a similar way and provide insights into the nature of the  **CNTF**  receptor complex.  Ref: 12707266 J Biol Chem< MedlineTA>J Biol Chem, 2003
- Cells that only express gp130 and LIF R  [ **LIFR** ] , but not  **CNTF**  R are refractory to stimulation by  **CNTF** .  Ref: 12765290 J Zhejiang Univ Sci< MedlineTA>J Zhejiang Univ Sci, 2003

**Non-interactive relationship** (e.g. studied together, co-existance, homology, etc.)

- ... at high concentrations, it can directly induce a signalling heterodimer of human gp130 and human  **LIFR**  in the absence of the  **CNTF**  receptor.  Ref: 12517918 J Biol Chem< MedlineTA>J Biol Chem, 2003
- Using RT PCR, expression of ciliary neurotrophic factor  [ **CNTF** ]  receptor alpha, leukemia inhibitory factor receptor  [ **LIFR** ] , and gp130 could be demonstrated in mRNA derived from murine corticotroph AtT 20 cells and murine pituitary tissue.  Ref: 12639901 Endocrinology, 2003
- Human  **CNTF**  elicits cellular responses by induction of a receptor complex consisting of the  **CNTF**  alpha receptor CNTFR, which is not involved in signal transduction, and the beta receptors gp130 and leukemia inhibitory factor receptor  [ **LIFR** ]   **LIFR** .  Ref: 12643274 J Biol Chem, 2003
- We concluded that the fusion protein Hyper  **CNTF**  had superagonistic activity on target cells expressing gp130 and LIF R  [ **LIFR** ] , but lacking membrane bound  **CNTF**  R.  Ref: 12765290 J Zhejiang Univ Sci< MedlineTA>J Zhejiang Univ Sci, 2003
- In order to investigate the effects of  **CNTF**  on fat cells, we examined the expression of  **CNTF**  receptor complex proteins  **LIFR** , gp130, and CNTFRalpha during adipocyte differentiation and the effects of  **CNTF**  on STAT, Akt, and MAPK activation.  Ref: 12424252 J Biol Chem, 2003
- We previously reported that the membrane distal cytokine binding domain CBD1 of  **LIFR**  could interact in vitro with soluble CNTFR sCNTFR in the absence of  **CNTF** .  Ref: 12707266 J Biol Chem< MedlineTA>J Biol Chem, 2003
- Human  **CNTF**  elicits cellular responses by induction of a receptor complex consisting of the  **CNTF**  a receptor CNTFR, which is not involved in signal transduction, and the b receptors gp130 and leukaemia inhibitory factor receptor  **LIFR** .  Ref: 12517918 J Biol Chem< MedlineTA>J Biol Chem, 2003
- Our studies clearly demonstrate that the expression of two of the three  **CNTF**  receptor complex components, CNTFRalpha and  **LIFR** , decreases during adipocyte differentiation.  Ref: 12424252 J Biol Chem, 2003
- ... at high concentrations, it can directly induce a signaling heterodimer of human gp130 and human  **LIFR**  in the absence of the  **CNTF**  receptor.  Ref: 12643274 J Biol Chem, 2003
- In addition, the expression of ciliary neurotrophic factor  [ **CNTF** ]  receptor CNTFR and leukemia inhibitory factor receptor  [ **LIFR** ]   **LIFR**  mRNAs, but not of interleukin 6 receptor mRNA, was up regulated after systemic lipopolysaccharide treatment.  Ref: 12127095 Neuroscience, 2002
- The proliferative results confirmed that  **CNTF**  required homodimerization of the gp130,  **CNTF**  R and LIF R  [ **LIFR** ]  receptor subunit whereas Hyper  **CNTF**  required heterodimerization of the gp130 and LIF R  [ **LIFR** ]  receptor subunit.  Ref: 12765290 J Zhejiang Univ Sci< MedlineTA>J Zhejiang Univ Sci, 2002
- Novel neurotrophin 1 B cell stimulating factor 3 is a recently cloned gp130 cytokine, acting through the tripartite ciliary neurotrophic factor  [ **CNTF** ]  receptor alpha leukemia inhibitory factor receptor  [ **LIFR** ]  gp130 receptor complex.  Ref: 12639901 Endocrinology, 2003
